# Genetic Structure Analysis of a Collection of Tunisian Durum Wheat Germplasm

**DOI:** 10.3390/ijms20133362

**Published:** 2019-07-09

**Authors:** Amine Slim, Luciana Piarulli, Houda Chennaoui Kourda, Mustapha Rouaissi, Cyrine Robbana, Ramzi Chaabane, Domenico Pignone, Cinzia Montemurro, Giacomo Mangini

**Affiliations:** 1 National Gene Bank of Tunisia, Boulevard du Leader Yasser Arafat Z. I Charguia 1, Tunis 1080, Tunisia; 2 SINAGRI S.r.l., Spin Off of the University of Bari Aldo Moro, Via Amendola 165/A, 70126 Bari, Italy; 3 Biotechnology and Physiology Laboratory, National Agronomic Research Institute of Tunisia (INRAT), Hedi Karray Street, Ariana 2049, Tunisia; 4 Institute of Biosciences and Bioresources of the National Research Council (IBBR-CNR), Via Amendola 165/A, 70126 Bari, Italy; 5 Department of Soil, Plant and Food Sciences (DiSSPA), Sect. Genetics and Plant Breeding, University of Bari Aldo Moro, Via Amendola 165/A, 70126 Bari, Italy

**Keywords:** durum wheat, population structure, genetic diversity, traditional varieties

## Abstract

The Tunisian durum wheat germplasm includes modern cultivars and traditional varieties that are still cultivated in areas where elite cultivars or intensive cultivation systems are not suitable. Within the frame of a collection program of the National Gene Bank of Tunisia (NGBT), durum wheat germplasm was collected from different Tunisian agro-ecological zones. The collected samples were studied using simple sequence repeats (SSRs) markers to explore the genetic diversity and evaluate the genetic structure in Tunisian germplasm. The results demonstrated significant diversity in the Tunisian durum wheat germplasm, with clear differentiation between traditional varieties and modern cultivars. The population structure analysis allowed the identification of five subpopulations, two of which appear to be more strongly represented in germplasm collected in central and southern Tunisia, where environmental conditions at critical development phases of the plant are harsher. Moreover these subpopulations are underrepresented in modern varieties, suggesting that traits of adaptation useful for breeding more resilient varieties might be present in central and southern germplasm. Moreover, our results will support, the activity of *in situ* on farm conservation of Tunisian durum wheat germplasm started by the National Gene Bank of Tunisia along with the *ex situ* approach.

## 1. Introduction

Durum (*Triticum turgidum* L. subsp. *durum*, genome AABB, 2n = 4× = 28) and common (*Triticum aestivum* L. subsp. *aestivum*, genome AABBDD, 2n = 6× = 42) wheats are species with significant agricultural importance as cereal grains throughout much of the world. In particular, durum wheat is grown mainly in the Mediterranean basin, and is primarily used for pasta, couscous and bread. Durum wheat, which originated in the Fertile Crescent, spread west of the Mediterranean basin, reaching the Iberian Peninsula and North Africa ca. 7000 years BC [1,2]. Wheat reached this area through Egypt, which acted as the bridge to the Fertile Crescent for the diffusion of wheat in North Africa. Other sources of wheat were maritime: wheat was introduced to Libya from Greece and Crete [3]. Moreover from the south of the Italian peninsula through the Sicily region, wheat reached the coasts of Tunisia, Morocco and Algeria [3]. This migration process, combined with both natural and human selection, led to the creation of local varieties along the Mediterranean basin that were well adapted to its different agro-ecological zones. Indeed, Tunisia and Algeria have a large collection of durum local varieties with great diversity, as observed for the first time by Boeuf in 1932 [4]. Contrasting environmental conditions of North Africa triggered a wide diversification of the durum wheat genome determining the development of several local populations. Thereby, North Africa is considered one of the principal secondary centers of diversification of durum wheat [5]. Studies on the origin and migration of durum wheat based on genetic diversity of landraces have shown that Tunisian landraces and those from the Mediterranean basin have a shared allelic identity and, consequently, that they originated from related seed sources and were exposed to similar environmental pressures and accumulated distinct mutations over time [6]. Durum wheat cultivation has continued to the present day as a staple food for local populations and possesses an outstanding socio-economical value as an important basic constituent of the diet in Maghreb countries [7,8].

In Tunisia during the 2018/2019 season, 0.5 M ha of durum wheat were cultivated, representing about 85% of the area devoted to wheat (common and durum), which in turn accounted for more than half of the cereal cultivated area [9]. The durum wheat germplasm cultivated includes about 45 varieties [10] grouped in (a) traditional varieties selected from Tunisian landraces (developed up to 1930s), (b) old cultivars produced by crossing Tunisian traditional varieties and foreign materials (developed up to 1970s) and (c) modern cultivars obtained in national and international breeding centers (developed up to present). The traditional varieties still prevail in rural areas where farmers still use traditional farming systems due to the impossibility of adopting modern intensive ones, or where this germplasm ensures tolerance to stresses [11,12]. The traditional varieties can be defined as the consequence of an empiric breeding program focused on exploiting the phenotypic variability among Tunisian durum landraces, such as ‘Biskri’, ‘Bidi’, ‘Mahmoudi’, ‘Chili’ and ‘Jenah Khottifa’ [7,8].

Several descriptions exist to define a ‘landrace’ [13,14]. Villa et al. [15] remarked that a landrace is ‘a dynamic population of a cultivated plant that has historical origin, distinct identity and lacks formal crop improvement, as well as often being genetically diverse, locally adapted and associated with traditional farming systems’. An autochthonous wheat landrace can be defined as a traditional variety with a high capacity to tolerate biotic and abiotic stresses, resulting in high yield stability and an intermediate yield level under a low input agricultural system [16]. Wheat landraces are valuable sources for broadening the genetic base of cultivated wheat. In fact, several studies using morphological, physiological and agronomic traits have shown that genetically diverse local germplasm well adapted to a broad range of environmental conditions can be considered an important reservoir of useful genes for exploitation in wheat breeding programs [17,18,19,20]. 

Molecular markers provide a satisfactory option for several genetic plant studies, since they are highly polymorphic and almost unlimited in number, do not vary among tissues or developmental stages of a plant and are not affected by environmental factors. Therefore, molecular markers can be used for genome analysis, and many of these have been applied with success for genetic mapping [21,22], cultivar identification [23] and diversity studies [19,20]. Different molecular markers have been developed (see review [24]). In particular, simple sequence repeats (SSRs) markers represent a useful alternative marker system because of their abundance in the wheat genome and easy detection as PCR-based molecular markers. Moreover, the advantages of SSRs include high information content, codominant inheritance, locus specificity and high reproducibility. Fifteen SSRs and five primer pair combinations of amplified fragment length polymorphism (AFLP) were tested by Medini et al. [10] in a wheat collection including Tunisian cultivars and wild wheat species. Cluster analysis separated the wild wheat species from the durum wheat cultivars, and these latter were further split in old and modern cultivars. The discriminant analysis carried out on 196 durum wheat lines issued from landraces collected in Tunisia and genotyped with diversity array technology sequencing (DArTseq) distinguished five groups that matched with the farmer’s variety nomenclature [25].

Nowadays, thanks to the activity of the National Gene Bank of Tunisia (NGBT), a program to collect durum wheat germplasm has been started. This program includes the idea of fostering the cultivation of traditional Tunisian durum germplasm by on farm conservation [26]. Within this framework, the maintenance and sustainable management of the genetic diversity present in autochthonous durum wheats are an absolute priority in order to preserve this genetic heritage, ensure food sovereignty and help smallholders cope with climate change. In this context the main goals of this work were to: (i) explore the genetic diversity in Tunisian durum wheat germplasm collected in different agro-ecological regions; (ii) evaluate the genetic structure of the Tunisian durum wheat genotypes; and (iii) clarify synonymy cases among the Tunisian durum wheat samples collected.

## 2. Results

### 2.1. Agro-Ecological Conditions of Tunisian Durum Wheat Zones 

**Figure 1:**
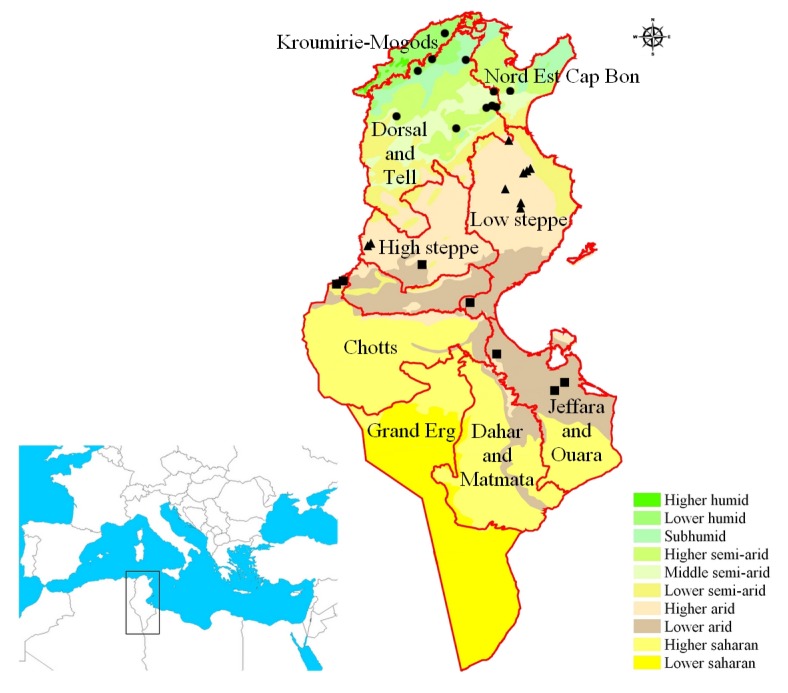
Agro-ecological map of Tunisia redrawn from CNEA [28]. The symbols ●, ▲ and ■ are used to indicate the location of sampling from the North, Center and South Tunisia, respectively.

The socio-agro-ecological zones of Tunisia follow a distribution similar to the natural regions (Figure 1). In the North, the agro-ecological zone of ‘Kroumirie and Mogods’ has an average yearly rainfall between 650 and 1500 mm, with severely reduced seasonal and annual variability. This area has two well-identified seasons: a wet season from October to April, and a dry season from June to September (Figure S1). In the Center, the agro-ecological zone of ‘Dorsal and Tell’ is formed by two different climatic units, due to the presence of mountain ridges that intercept the humid currents from the Mediterranean (Figure 1). This is a semi-arid bio-climate with cool and temperate winters and precipitations varying with the elevations (higher in the mountain area and lower moving East). The South is characterized by an arid Mediterranean climate with a long dry season (8–9 months) and rainfall ranging from 100 to 200 mm (Figure S1). 

In these three zones, the production of durum wheat shows a strong North-to-South decline along the cline determined by the bio-climate of the areas, as reported by Latiri et al. [27]. The durum wheat germplasm utilized in the present study is representative of the three agro-ecological zones previously described.

### 2.2. Genetic Diversity 

A set of 16 selected simple sequence repeats (SSR) markers were used to study the genetic diversity in a durum wheat collection including 41 Tunisian samples and 13 cultivars (Table 1). A total of 95 alleles was obtained, ranging from 13 (*Xgwm193*) to 2 (*Xgwm154*). The polymorphism information content (PIC) was used to evaluate the informativeness of SSRs. The PIC mean value of the 16 SSRs was 0.57 with a range from 0.82 (*Xgwm193*) to 0.28 (*Xwmc406*) as reported in Table 1. A strong relationship (*r* = 0.84***; *p* < 0.001) was observed between the number of alleles and PIC values. The Shannon’s information index (*I*) value ranged from 2.07 (*Xgwm193*) to 0.51 (*Xwmc406*). The expected heterozygosity (*He*), which corresponds to heterozygosity at a single locus, ranged from 0.28 (*Xwmc406*) to 0.82 (*Xgwm193*). The fixation index (*F*), which compares the expected heterozygosity (*He*) with observed heterozygosity (*Ho*) to estimate the degree of allelic fixation, ranged from –0.30 (*Xgwm154*) to 1.00 (*Xbarc45* and *Xgwm193* having all the alleles fixed) (Table 1). The *F* mean value was found to be 0.82 for the whole collection, and *F* values were positive in 15 SSR markers, revealing that the majority of the alleles were fixed.

**Figure 2:**
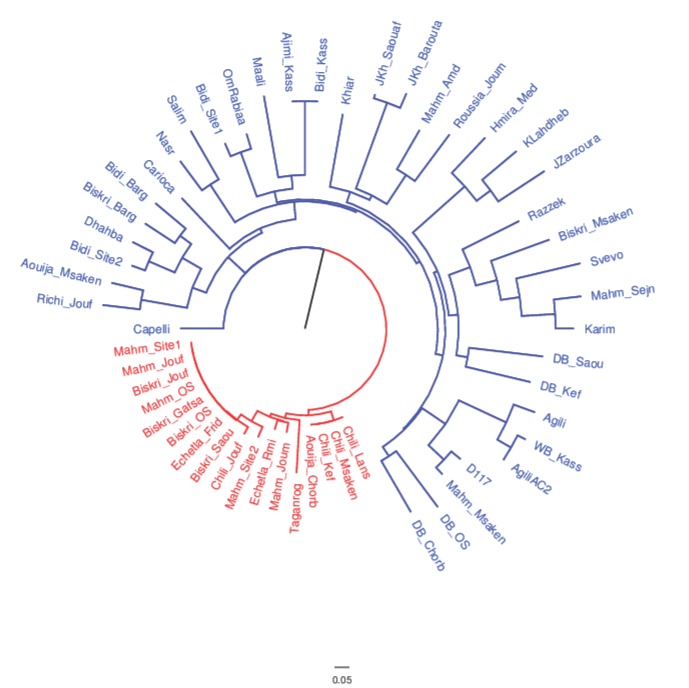
Dendrogram of durum wheat collection resulting from the neighbor-joining cluster analysis based on the dissimilarity matrix obtained from SSR allelic data. The colors blue and red were used to distinguish the two main clusters.

Based on the dissimilarity matrix, a neighbor-joining dendrogram clearly showed the presence of two main clusters (Figure 2). The first cluster included 24 Tunisian durum wheat samples and 12 cultivars. This group included nine Tunisian durum wheat cultivars, suggesting a genetic relationship among them. Indeed, Karim and Razzak were the parental lines of several modern durum cultivars obtained from the breeding programs of the National Agronomic Research Institute of Tunisia (INRAT). The second cluster incorporated mainly Tunisian samples and the cultivar Taganrog. In this cluster, the group constituted by four Tunisian durum samples, (Chili_Lans, Chili_Msaken, Chili_Kef and Aouija_Chorb) was found to be genetically identical. This result suggests that, probably, the name ‘Aouija’ could have been erroneously attributed by local farmers to Chili, although morphologically different. Moreover, four Tunisian durum samples named Biskri_Saou, Biskri_OS, Biskri_Jouf and Biskri_Gafsa were found to be genetically identical and undistinguishable from three samples named Mahmoudi_OS, Mahmoudi_Jouf and Mahmoudi_Site1, thus suggesting a putative case of synonymy. The two main clusters identified in the dendrogram were used to calculate the AMOVA, revealing that 22% of the total variation was found among the clusters while the rest (78%) was within clusters (Table 2). Finally, cluster analysis distinguished the samples with white glumes and black awns from the rest of samples. The durum wheat samples with these traits are included in the second cluster, confirming that the landraces Mahmoudi, Biskri and Chili were similar in these traits [8].

### 2.3. Genetic Structure

For population genetic structure analysis, Bayesian clustering modeling was performed using STRUCTURE software with genotyping data generated according to 16 SSR markers. As the clustering model presumes the underlying existence of K clusters, an Evanno [29] test was performed and yielded K = 5 as the highest log-likelihood (Figure S2). In this way, the durum wheat collection showed a genetic structure split into five subpopulations. A durum wheat sample was considered belonging to a subpopulation if its membership coefficient (*Q*) was > 0.60, as suggested by Chen et al. [30]. The five subpopulations obtained by the Bayesian method were used to calculate the AMOVA, revealing that 37% of the total variation was found among the subpopulations, while the rest of variation (63%) was within subpopulations (Table 3). 

**Figure 3:**
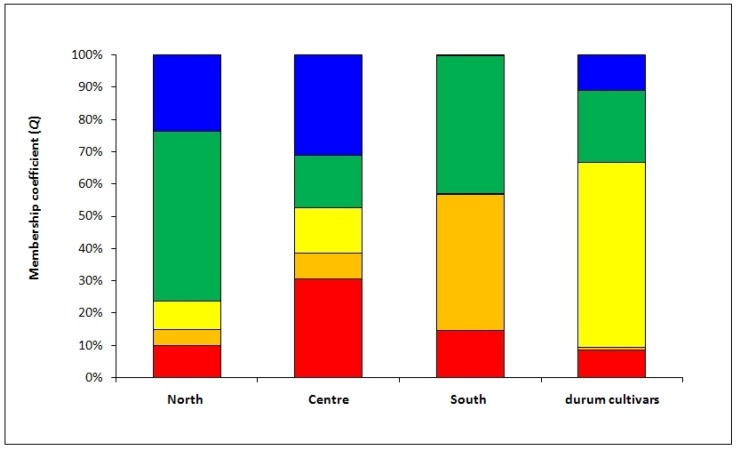
Membership coefficient (*Q*) mean of the Tunisian durum samples split according to the agro-ecological zones of sampling location (North, Center and South) and modern durum cultivars. The different colors indicate the five subpopulations detected using a Bayesian approach (red: subpopulation 1; orange: subpopulation 2; yellow: subpopulation 3; green: subpopulation 4 and blue: subpopulation 5).

The collection was divided in four groups: three included the Tunisian durum wheat samples, spilt according to the agro-ecological zones of sampling location (North, Center, and South); the fourth group included the modern durum cultivars (Figure 3). In each group, the mean *Q* of the five subpopulations, detected by structure analysis, was calculated and showed a strong genetic stratification. In particular, two main subpopulations (in orange and red) were relatively abundant in Tunisian durum samples, but rare (<10%) in modern durum cultivars. Two subpopulations (in orange and green) were found the main genetic components (some 80%) of durum wheat samples collected in the South of Tunisia. A subpopulation (in yellow) was present at a frequency >60% in modern durum cultivars and almost absent (<1%) in durum wheat samples collected in the South of Tunisia. The AMOVA confirmed that 9% of the total variation was found among Tunisian durum samples split according agro-ecological zones, while the rest (91%) was within groups (Table 4).

## 3. Discussion

During the second half of the last century, a significant increase in wheat yield was observed in Tunisia. This was a consequence of the introduction of modern high-yielding varieties able to optimize water and soil resources, as well as a result of improvements in agronomic techniques, such as better crop management [7,8]. 

In Tunisia, as in the whole Mediterranean region, rainfall is highly variable in its yearly distribution [27]. The overall yearly rainfall is differently distributed over the country, with northern regions much wetter than the Center and South (Figure 1 and Figure S1). The growing season of wheat, with sowing between November and December, extends through the wet and cool season when in central/southern regions there is a reduced level of precipitation but evaporation is relatively low, thus securing enough water for the plants’ needs. More critical is the period between April and May, the period of flowering-to-grain filling, when high temperatures or drought stress can dramatically reduce kernel setting, and thus yield [22,27,31]. In this period, the difference in precipitation among the three regions of Tunisia drastically increases (Figure S1). Therefore, it is possible to suppose that plants adapted to central and principally southern environments should possess traits of adaptation, allowing them to escape from these limiting environmental conditions.

In our study, a set of 16 SSR markers with a good coverage of the A and B genomes of durum wheat were tested in the composed durum wheat collection. They allowed the identification of 95 alleles, with an average of 5,9 alleles/SSR locus. The considerable number of alleles was due to the multi-allelic nature of these markers that facilitates variability detection [32]. Similar results were reported by Chen et al. [30] in a Chinese winter wheat collection, although a large range of alleles per locus (4.5–18.1) was reported in the literature for a wider germplasm collection [33,34]. The genetic diversity of the collection (*He* = 0.58) was similar to the values reported by previous studies involving durum wheat collections (*He* values between 0.55 and 0.68) [33,35]. 

To define the genetic distances among samples, the matrix obtained with a simple matching coefficient was also used to produce a neighbor-joining dendrogram (Figure 2). This dendrogram clearly divided collected germplasm into two distinct groups: the one hosting only traditional varieties, (mostly Biskri, Mahmoudi and Chili), and one with a more assorted composition (including both traditional and modern varieties). In this latter group, the distances separating the entries are much higher than in the former one, which we will refer to as the ‘core traditional varieties’ (CTVs) group.

To better investigate this result, an analysis of the molecular variance was performed to test different sources of variation. In the first instance, we tested the distribution of molecular variation among the CTV group and the rest of the analyzed germplasm. The results clearly indicate that the CTV group is unquestionably differentiated from the rest of the analyzed material, since 22% of the total variation is between the two groups of samples (Table 2). In a second run, three groups were tested: the CTV, the one formed by entries described by farmers as traditional varieties and, finally, modern cultivars. In this case, AMOVA demonstrated that the distribution of the molecular variance conformed to the grouping, even though at a lower extent than in the former analysis (Table S1).

Nevertheless, the above analyses did not provide any information on the distribution of genotypes along the geographic cline. To this end a Bayesian approach was utilized by analyzing genotypic data through the STRUCTURE software. Structure analysis assigns each genotype to a specific subpopulation and five different ones were identified after the Evanno test [29]. The robustness of these results was confirmed by the AMOVA used to test the distribution of variation among and within these five subpopulations. In fact, the AMOVA indicated that 37% of the total variation observed was among these five groups (Table 3). After assigning each genotype to a specific subpopulation, as described in the results section, we tested the richness of each subpopulation using the three collection zones (North, Center, and South) as discriminating factors for Tunisian samples; a fourth discriminating factor was the modern durum cultivars (see Figure 3). 

The approach described above allowed us to observe that some genotypes (belonging to the subpopulations indicated with the colors red and orange in Figure 3) were increasingly frequent along the North–South cline, while at the same time the modern cultivars tended to be decreasingly represented. Conversely, in the modern cultivars, the presence of traditional germplasm was scarce. 

This picture indicates that: (a) modern varieties are more fit for cultivation in the northern part of Tunisia, where the rainfall is adequate for new high-yielding varieties that develop their full potential only in the presence of moderate to high inputs; (b) there is an increased presence of traditional varieties and CTVs in the Center and South groups, which are characterized by reduced and more erratic precipitations. 

These findings indicate that the traditional varieties, originally developed from North African landraces, might possess traits of resistance to unfavorable climatic conditions that are absent in the modern cultivars. This is not surprising since landraces and derived traditional varieties are the result of a non-scientific process of selection driven by farmers’ cultural values and by natural adaptation to local environment [13]. 

The fact that some old cultivars, such as AgiliAC2, are still called by the names of traditional varieties might be the result of different causes. In fact, the improved material of the 1970s–1990s might have been distributed using the names of the traditional varieties in order to overcome the diffidence of more traditional farmers against innovations. Another reason might reside in a phenotypic resemblance of some morphological traits of the new introductions with those of the CTVs, or by the misnaming of landraces during seed exchange among farmers or during the collecting missions undertaken by the NGBT [25].

In conclusion, our results showed that the molecular markers distinguished Tunisian durum wheat germplasm (named the CTV group) and detected putative synonymy and homonymy cases. These results could be useful to increase *ex situ* conservation efficiency, because SSRs allow the detection of redundant duplications within durum germplasm conserved in the NGBT. *In situ* on-farm conservation, in addition to the *ex situ* approach, could ensure the long-term conservation of genetically diverse Tunisian durum wheat available in local germplasm, which is a reservoir of genes for drought, heat and salinity tolerance and quality traits. The traditional varieties could help maintain the use of local knowledge and challenge ongoing genetic erosion due to the spread of modern pure line cultivars.

The population structure analysis suggests a strong genetic stratification along the North–South cline of Tunisia. This could be the result of breeding programs from the second half of the last century that produced high-yielding varieties for the northern highly productive areas rather than tackling the agro-ecological conditions of the Center and South.

The strategy of incorporating adaptation traits found in the genetic diversity pool of landraces and traditional varieties into modern breeding programs is widely supported [36]. In this way it will also be possible to contend with the challenges imposed by climate change and the survival of agriculture in marginal lands [36,37,38]. Correct and scientifically driven programs aiming at *in situ* conservation of agro-biodiversity on farms are essential, particularly for those species that are the food staple of Mediterranean populations and Tunisians in particular, such as durum wheat [39]. In fact, the end-use caracteristics are among the selection drivers used by past farmers during the non-scientific selection of traditional landraces. This explains the preference of genotypes providing better couscous or more valuable tabouna (a traditional sort of bread).

## 4. Materials and Methods 

### 4.1. Plant Material

A durum wheat collection including 41 Tunisian samples (Table 5) and 13 cultivars (Table 6) were considered in this study. The Tunisian durum samples, named as indicated by the farmers, were collected during 2012–2018 (Figure 1) and conserved by the National Gene Bank of Tunisia (NGBT). The Tunisian durum cultivars (9), and French durum cultivar (Carioca) were obtained from NGBT, while the three Italian durum cultivars (Cappelli, Svevo and Taganrog), used as references, were obtained from the Department of Soil, Plant and Food Sciences (DiSSPA), Sect. Genetics and Plant Breeding, University of Bari Aldo Moro (Italy). 

The data of monthly rainfall were obtained from the National Institute of Meteorology of Tunisia and derived from the mean of the last 30 years (1988–2018). The data were recorded at three stations in the North (Bizerte, Tunis and Tabarka), three stations in the Center (Kairouan, Monastir and Sfax) and two stations in the South (Djerba and Tozeur).

### 4.2. DNA Extraction and SSR Assays

The genomic DNA was extracted from 3 g of fresh young leaves from a single plant for each sample using the cetyltrimethyl ammonium bromide (CTAB) method [44]. The DNA quality was evaluated in agarose gel (0.8%) (Biomatik, Wilmington, DE, USA) and quantified using NanoDropTM ND2000 (Thermo Scientific, MA, USA). All samples were normalized to a standard concentration of 50 ng by adding HPLC-grade water (Sigma Aldrich, St. Louis, MO, USA). The durum wheat collection was evaluated with a set of 16 simple sequence repeats (SSRs) markers (reported in Table 1). The SSRs were selected based on the consensus map of tetraploid wheat [45] and following criteria proposed by Laidò et al. [43]: locus-specific amplification, low complexity, robust amplification and good genome coverage (nearly one marker for each chromosome arm). The sequence primers were available in the GrainGenes database [46]. The amplification reactions were performed in a final volume of 20 μL, containing the template DNA (50 ng) forward primer with the M13 (−21bp) tail at its 5′ end (0.06 μM), reverse primer (0.16 μM), M13 primer labeled with 6-Carboxyfluorescein (6-FAM) (0.05 μM) or 2’-chloro-5’-fluoro-7’,8’-fused phenyl-1.4-dichloro-6-carboxyfluorescein (NED) (0.10 μM), or 2′-chloro-7′phenyl-1,4-dichloro-6-carboxy-fluorescein (VIC) (0.05 μM), dNTPs (2 mM), buffer (1×) and Taq DNA polymerase (0.6 U). PCR reactions were performed in a thermal cycler (Bio-Rad Laboratories, Hercules, CA, USA) as follows: an initial denaturing step at 94 °C for 3 min, followed by 35 cycles of 94 °C for 1 min, 59 °C for 1 min, 72 °C for 2 min and a final extension step at 72 °C for 7 min. In order to verify the PCR efficiency, the PCR fragments were separated on 2.0% agarose gels containing Nancy-520 DNA Gel Stain (Sigma Genosys, St. Louis, MO, USA), and visualized under UV light. The amplification products were detected by the automatic capillary sequencer ABI PRISM 3100 Genetic Analyzer (Applied Biosystems, Waltham, MA, USA), and the sample analyses were carried out with GeneMapper genotyping software version 5.0 (Figure S3). The internal molecular weight standard was GeneScanTM 600 LIZ dye Size Standard (ThermoFisher Scientific, Waltham, MA, USA).

### 4.3. Data Analysis 

The genetic indices as number of alleles (*Na*), Shannon’s information index (*I*), heterozygosity observed (*Ho*), heterozygosity expected (*He*) and fixation index (*F* = *He − Ho/He*) were calculated using the GenAlEx program version 6.5 [47,48]. The informativeness of SSR markers was measured by the polymorphism information content (PIC) according to Weber [49] and Anderson et al. [50]. The allelic data was used to obtain the dissimilarity matrix using the simple matching coefficient by DARwin program version 6.01 [51]. This dissimilarity matrix was utilized for constructing the dendrogram using the neighbor-joining algorithm [52]. The confidence interval of the genetic relationships among the samples was determined by 1000 bootstraps.

The molecular data were processed using the STRUCTURE program version 2.3.4 [53]. The number of sub-populations (K) was estimated by 20 independent runs for each K (from 2 to 20) applying the admixture model, 100,000 Markov Chain Monte Carlo (MCMC) repetitions and a 100,000 burn-in period. The means of the log-likelihood estimates for each K were calculated. The true K was determined using the Evanno ΔK [29] using STRUCTURE HARVESTER [54,55]. Analysis of molecular variance (AMOVA) was used to partition the genetic variation into inter- and intra-gene pool diversities in the durum wheat collection using GenAlEx program version 6.5 with 1000 permutations.

## Figures and Tables

**Table 1:** Summary of genetic variation detected in the durum wheat collection tested with 16 SSR markers.

Locus	Chromosome Arm	Repeat Motif	Allele Size Range	*Na*	PIC	*I*	*Ho*	*He*	*F*
*Xgwm633*	1AS	not available	131–151	6	0.60	1.22	0.02	0.60	0.97
*Xgwm124*	1BL	(CT)_27_(GT)_18_ imp	208–224	5	0.36	0.72	0.02	0.36	0.95
*Xwmc406*	1BS	(CA)_16_	225–237	3	0.28	0.51	0.06	0.28	0.79
*Xgwm311*	2AL	(GA)_29_	132–176	10	0.77	1.83	0.09	0.77	0.89
*Xgwm299*	2BL	(GA)_31_(TAG)_4_	204–266	5	0.61	1.19	0.12	0.61	0.81
BQ170801^1^	2BS	(AC)_n_	130–138	3	0.35	0.62	0.02	0.35	0.95
*Xbarc45*	3AS	(TAA)_10_	193–233	6	0.64	1.28	0.00	0.64	1.00
BJ274952^1^	3BS	(ACGAT)_n_	170–176	3	0.51	0.74	0.04	0.51	0.93
*Xwmc262*	4AL	(GA)_29_	163–209	6	0.64	1.31	0.10	0.64	0.84
*Xgwm495*	4BL	(GA)_20_	174–192	7	0.72	1.55	0.02	0.72	0.97
*Xgwm154*	5AS	(GAT)_37_ imp	162–174	2	0.36	0.54	0.46	0.36	−0.30
*Xgwm408*	5BL	(CA)_>22_(TA)(CA)_7_(TA)_9_	162–196	4	0.50	0.89	0.21	0.50	0.58
*Xwmc235*	5BL	(CA)_25_	239–251	5	0.68	1.24	0.06	0.68	0.91
*Xgwm427*	6AL	(CA)_31_(CA)_22_	200–244	7	0.62	1.32	0.08	0.62	0.87
*Xgwm193*	6BS	(CT)_24_ imp (CA)_8_	174–206	13	0.82	2.07	0.00	0.82	1.00
*Xgwm537*	7BS	(CA)_18_(TA)_13_	211–256	10	0.76	1.75	0.04	0.76	0.95

Expressed sequence tag; PIC = polymorphism information content; *Na* = number of alleles, *I* = Shannon’s information index; *Ho* = heterozygosity observed; *He* = heterozygosity expected; *F* = fixation index.

**Table 2:** Analysis of molecular variance (AMOVA) of durum wheat collection subdivided into the two main groups and detected using the neighbor-joining algorithm in DARwin software version 6.01.

Source of Variation	df	SS	MS	Est. Var.	%	*p* Values
Among groups	1	122.57	122.57	4.56	22	<0.001
Within groups	52	852.23	16.39	16.39	78	
Total	53	974.80		20.95	100	

df = degree of freedom; SS = sum of squares; MS = mean squares; Est. var. = estimate of variance; % = percentage of total variation.

**Table 3:** Analysis of molecular variance (AMOVA) of durum wheat collection split according to genetic structure estimated using Bayesian approach in STRUCTURE software version 2.3.4.

Source of Variation	df	SS	MS	Est. Var.	%	*p* Values
Among subpopulations	4	347.26	86.82	7.31	37	<0.001
Within subpopulations	52	607.03	12.65	12.65	63	
Total	53	954.28		19.95	100	

df = degree of freedom; SS = sum of squares; MS = mean squares; Est. var. = estimate of variance; % = percentage of total variation.

**Table 4:** Analysis of molecular variance (AMOVA) of durum wheat collection split according to the eco-geographical zones in which they were collected (North, Center, South).

Source of Variation	Df	SS	MS	Est. Var.	%	*p* Values
Among eco-geographical groups	3	113.98	37.99	1.61	9	<0.001
Within eco-geographical groups	50	860.84	17.22	17.22	91	
Total	53	974.80		18.83	100	

df = degree of freedom; SS = sum of squares; MS = mean squares; Est. var. = estimate of variance; % = percentage of total variation.

**Table 5:** List of Tunisian durum wheat samples included in the collection.

ID	Local Name	Agro-ecological Zone	Governorate	Location	Latitude (N)	Longitude (E)
NGBT001	Mahm_Amd	North	Beja	Ain Rihana	36.8699	9.074
NGBT002	Mahm_Joum		Bizerte	Kef Zoraâ	36.3147	10.0844
NGBT003	Roussia_Joum		"	Kef Zoraâ	36.9932	9.2262
NGBT004	Mahm_Sejn		"	Sejnane	37.1483	9.375
NGBT005	Chili_Kef		Kef	Eddir	36.2181	8.7689
NGBT006	DB_Kef		"	Eddir	36.2181	8.7689
NGBT007	Chili_Lans		Mannouba	Lansarine	36.8337	9.6598
NGBT008	Biskri_Barg		Siliana	Marjaâ Aouam	36.0869	9.5344
NGBT009	Bidi_Barg		"	Marjaâ Aouam	36.4003	10.1498
NGBT010	Biskri_Jouf		Zaghouan	El Jouf	36.2999	10.113
NGBT011	Chili_Jouf		"	El Jouf	36.3152	10.0854
NGBT012	Mahm_Jouf		"	El Jouf	35.6625	10.4881
NGBT013	Richi_Jouf		"	El Jouf	36.9932	9.2262
NGBT014	Biskri_OS		"	Oued Sbaihya	36.3059	10.1157
NGBT015	DB_OS		"	Oued Sbaihya	36.4003	10.1498
NGBT016	Mahm_OS		"	Oued Sbaihya	36.4003	10.1498
NGBT017	Mahm_Site2		"	Oued Sbaihya	36.4961	10.2321
NGBT018	Biskri_Saou		"	Saouaf	36.4003	10.1498
NGBT019	DB_Saou		"	Saouaf	36.291	10.1514
NGBT020	JKh_Saouaf		"	Saouaf	36.291	10.1514
NGBT021	Mahm_Site1		"	Saouaf	36.291	10.1514
NGBT022	JKh_Barouta	Center	Kairouan	Barrouta	36.291	10.1514
NGBT023	D117		"	Koudiat Sisseb	35.4128	10.1615
NGBT024	Ajimi_Kass		Kasserine	Om Lagsab	35.9776	10.2145
NGBT025	Bidi_Kass		"	Om Lagsab	34.8119	8.3764
NGBT026	WB_Kass		"	Om Lagsab	34.8119	8.3764
NGBT027	Aouija_Chorb		Mahdia	Chahda Est	34.8142	8.3842
NGBT028	Bidi_Site1		"	Chahda Est	35.3175	10.3681
NGBT029	Bidi_Site2		"	Chahda Est	35.3175	10.3681
NGBT030	DB_Chorb		"	Chahda Est	35.3092	10.3681
NGBT031	Aouija_Msaken		Sousse	Knaies	35.3175	10.3678
NGBT032	Biskri_Msaken		"	Knaies	35.6394	10.4734
NGBT033	Chili_Msaken		"	Knaies	35.6449	10.4792
NGBT034	Mahm_Msaken		"	Knaies	35.6428	10.4747
NGBT035	JZarzoura	South	Gabès	Matmata	33.6303	10.0517
NGBT036	KLahdheb		"	Menzel Habib	34.1989	9.7092
NGBT037	Biskri_Gafsa		Gafsa	Ouled Whiba	34.6675	9.088
NGBT038	Hmira_Med		Médenine	Chahbania	33.2461	10.8281
NGBT039	Agili		"	Dkhilet Esstout	33.3239	10.9239
NGBT040	Echetla_Frid		Tozeur	El Frid	34.4631	8.0477
NGBT041	Echetla_Rmi		"	Rmitha	34.4266	7.9858

**Table 6:** Durum wheat cultivars included in the collection.

Cultivar	Origin	Pedigree
Agili AC2 ^a^	Tunisia	selected from Agili landrace
Karim ^b^	Mexico/Tunisia	Jori-69(SIB)/(SIB)Anhinga//(SIB)Flamingo
Khiar ^b^	Mexico/Tunisia	Chen(SIB)//Rus(SIB)/(SIB)Flamingo//Mexicali-75/3/Shearwater
Maâli ^b^	Tunisia	CMH80A.1046/4TTURA/CMH77//CMH77.774/3YAV79/5/Razzak/6
Nasr ^b^	Tunisia/Syria	GdoVz512/Cit//Ruff/Fg/3/Pin/Gre//Trob ICD85-1340-ABL-0TR-10b-3b-0b
Razzak ^b^	Tunisia	DMx69-331/Karim
Salim ^b^	Tunisia	Altar84/FD84-19-126-1-2/Razzak/3/Krf/BaladiaHamra D93-21-2b-9b-0b-2b-6b-0b
Dhahbi ^c^	Tunisia	Karim/4/BD2337//D68-8-6A-3A/Karim’’S’’/3/Src2/Src1
OmRabia ^a^	Tunisia/Syria	JO/Haurani
Carioca ^d^	France	CID 479402
Cappelli ^e^	Italy	selected from Jenah Khottifa landrace
Svevo ^e^	Italy	CIMMYT’s selection/Zenit
Taganrog ^e^	Italy	selected from Taganrog landrace

^a)^ [8]; ^b)^ [40]; ^c)^ [41]; ^d)^ [42]; ^e)^ [43].

## Supplementary Materials

Supplementary materials can be found at https://www.mdpi.com/1422-0067/20/13/3362/s1. Figure S1: Monthly rainfall (mm) in the three Tunisian macro areas: North (in green), Center (in red) and South (in yellow). Figure S2: Delta ΔK for differing numbers of subpopulations (K) estimated in durum wheat collection with 16 SSR markers. Table S1: Analysis of molecular variance (AMOVA) of durum wheat collection split in three groups: Core Traditional Varieties (CTVs), entries named as traditional varieties by farmers and durum cultivars. Figure S3: Electropherogram of Tunisian durum wheats D117 and Agili AC2 tested with *Xgwm193* SSR marker labeled with fluorescent dye NED. The alleles 182 and 186 bp were found in D117 and Agili AC2 durum samples, respectively.

## Abbreviations

NGBT	National Gene Bank of Tunisia
CTAB	CetylTrimethyl Ammonium Bromide
SSR	Simple Sequence Repeat
*Na*	Number of alleles
*I*	Shannon’s information index
*Ho*	Heterozygosity observed
*He*	Heterozygosity expected
*F*	Fixation index
PIC	Polymorphism Information Content
MCMC	Markov Chain Monte Carlo
AMOVA	Analysis of molecular variance
CTV	Core Traditional Varieties
