# Supplementary material for: Genetic Structure Analysis of a Collection of Tunisian Durum Wheat Germplasm
Source: Int J Mol Sci. 2019 Jul 9;20(13):3362. doi: 10.3390/ijms20133362 (PMC6651592; doi:10.3390/ijms20133362)
Supplement: Supplementary file 1 [file ijms-20-03362-s001.zip › Supplementary materials ijms-523404/Figure S1.pdf]

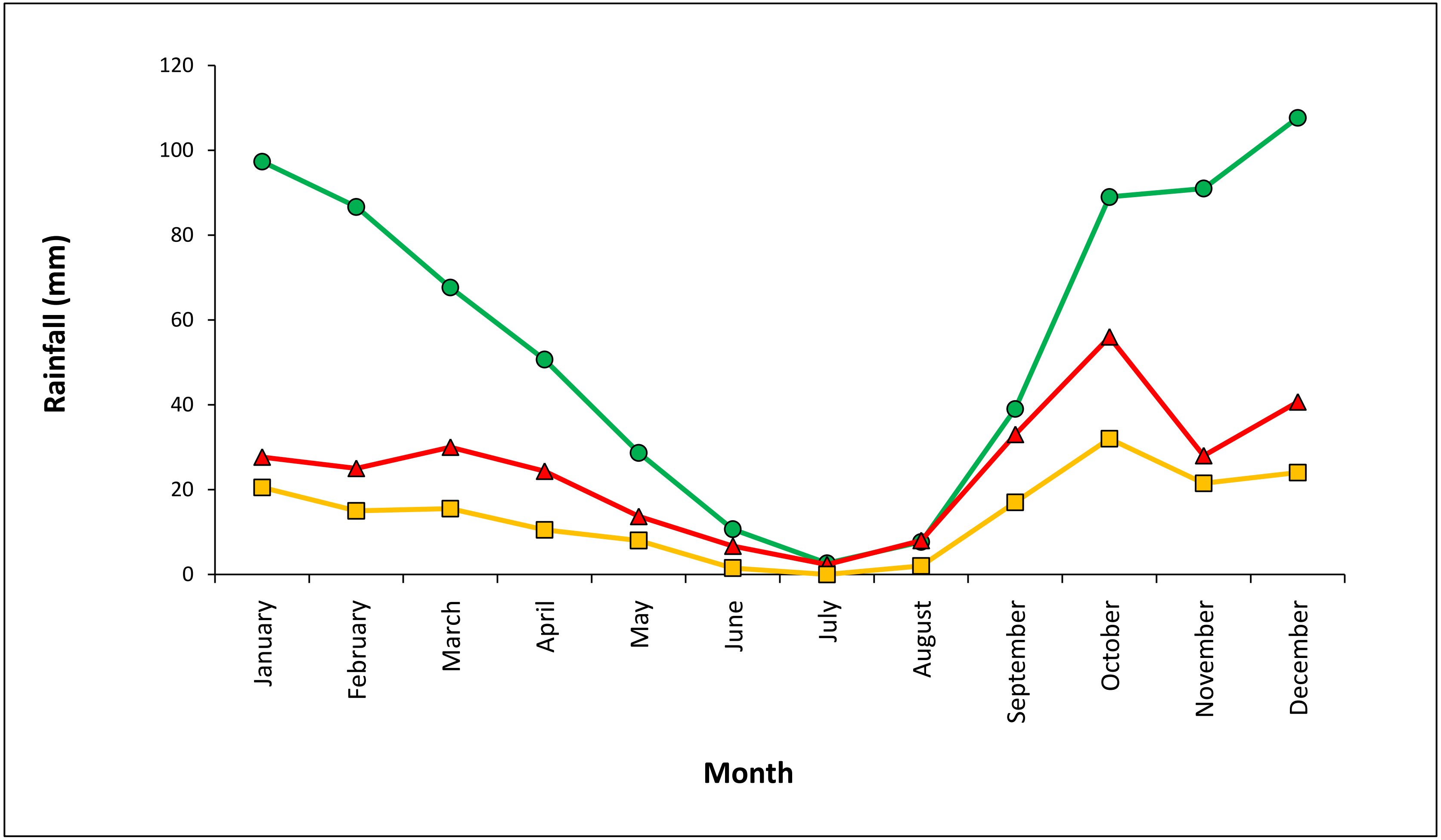

Figure S1: Monthly rainfall (mm) in the three Tunisian macro areas: North (in green), Center (in red) and South (in yellow).
