# Supplementary material for: Genetic Structure Analysis of a Collection of Tunisian Durum Wheat Germplasm
Source: Int J Mol Sci. 2019 Jul 9;20(13):3362. doi: 10.3390/ijms20133362 (PMC6651592; doi:10.3390/ijms20133362)
Supplement: Supplementary file 1 [file ijms-20-03362-s001.zip › Supplementary materials ijms-523404/Table_S1.pdf]

Table S1: Analysis of molecular variance (AMOVA) of durum wheat collection split in three groups: Core Traditional Varieties (CTVs), entries named as traditional varieties by farmers and durum cultivars.

Source of variation	df	SS	MS	Est. Var.	%	*P* values
Among groups	2	158.41	79.21	3.67	19	<0.001
Within groups	51	816.39	16.01	16.01	81	
Total	53	974.80		19.68	100	

df = degree of freedom, SS=sum of squares, MS=mean squares, Est. var. = estimate of variance, % = percentage of total variation.
